# Usefulness of Bacterial Culture of Drainage Fluid for Predicting Surgical Site Infection After Crohn’s Disease Surgery

**DOI:** 10.1002/ags3.12530

**Published:** 2021-11-27

**Authors:** Momoko Ichihara, Takayuki Ogino, Makoto Fujii, Naotsugu Haraguchi, Hidekazu Takahashi, Norikatsu Miyoshi, Mamoru Uemura, Yuichiro Doki, Hidetoshi Eguchi, Tsunekazu Mizushima

**Affiliations:** ^1^ Department of Gastroenterological Surgery Osaka University Graduate School of Medicine Osaka Japan; ^2^ Department of Therapeutics for Inflammatory Bowel Diseases Osaka University Graduate School of Medicine Osaka Japan; ^3^ Division of Health Science Osaka University Graduate School of Medicine Osaka Japan

**Keywords:** bacterial culture, CD, drainage fluid, SSI

## Abstract

**Aim:**

Early detection of surgical site infection (SSI) allows for appropriate management after Crohn's disease (CD) surgery. The aim of this study was to evaluate the usefulness of bacterial culture of postoperative drainage fluid after CD surgery.

**Methods:**

This study included 110 patients with CD who underwent surgery with bowel resection between January 2010 and March 2020 at Osaka University Hospital. Patients with only perianal surgery or incomplete records were excluded. Risk factors for SSI were evaluated in the context of clinical findings, including bacterial culture of postoperative drainage fluid, and bacterial species related to SSI were also examined.

**Results:**

Of 110 patients, 18 (16.4%) developed SSI. Organ/space SSI developed in six, and a positive bacterial culture of drainage fluid (D‐Posi) was found in five (83.3%). Of 104 patients without organ/space SSI, 31 (29.8%) were D‐Posi (*P* = .027). Similarly, 68.8% with incisional SSI were D‐Posi, whereas 26.6% without incisional SSI were D‐Posi (*P* = .0021). Multivariate analysis revealed that D‐Posi was an independent risk factor in both organ/space and incisional SSI. Bacterial examination showed that *Pseudomonas aeruginosa* and *Enterococcus faecalis* were significantly detected in patients with SSI.

**Conclusion:**

This study suggests the usefulness of postoperative drainage fluid bacterial culture for early diagnosis of SSI after CD surgery.

## Graphical abstract

We demonstrate that bacterial culture of postoperative drainage fluid was useful to predict surgical site infection in the early phase after Crohn's disease surgery. This study suggests that bacterial culture allowed accurate diagnosis of SSI and prevention of exacerbations. Also, bacterial examination showed that *Pseudomonas aeruginosa* and *Enterococcus faecalis* were significantly detected in patients with SSI.

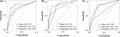

## 1 INTRODUCTION

Crohn's disease (CD) involves chronic intestinal inflammation and differences in gut bacterial species compared with healthy controls.^1^, ^2^ Despite improvements in medical treatments, 70%–90% of patients will undergo surgery in their lifetime.^3^ More than half of them will require surgical intervention within 15 y of previous operations because of recurrence or new lesions occurrence.^4^, ^5^, ^6^ Postoperative complications may not only delay resumption of medication but also trigger recurrence and complicate subsequent surgeries because of adhesions.^7^, ^8^ If complications are detected in the early phase, exacerbations could be avoided and prognosis improved.^7^, ^8^ For these reasons, accurate predictors of complications are needed.

Surgical site infection (SSI) is one of the postoperative complications, and the rate of SSI after CD surgery is higher than after colorectal cancer surgery.^4^, ^9^, ^10^, ^11^ Several SSI risk factors have been identified in patients with CD, including anemia, a longer duration of surgery, and higher intraoperative lactate level.^4^, ^11^ Although these are indirect risk factors for SSI, a direct factor is bacterial contamination of the surgical site.^12^ Few reports, however, have described the association between positive bacterial culture of lavage or drainage fluid and SSI in gastrointestinal surgery.^12^, ^13^, ^14^ To our knowledge, no studies have evaluated the role of positive bacterial culture of drainage fluid (D‐Posi) after CD surgery.

We hypothesized that D‐Posi could predict SSI in the early phase after CD surgery. The aims of this study were to evaluate the clinical impact of D‐Posi in detecting SSI and the characteristics of detected bacterial species after CD surgery.

## 2 METHODS

### 2.1 Patients

**FIGURE 1:**
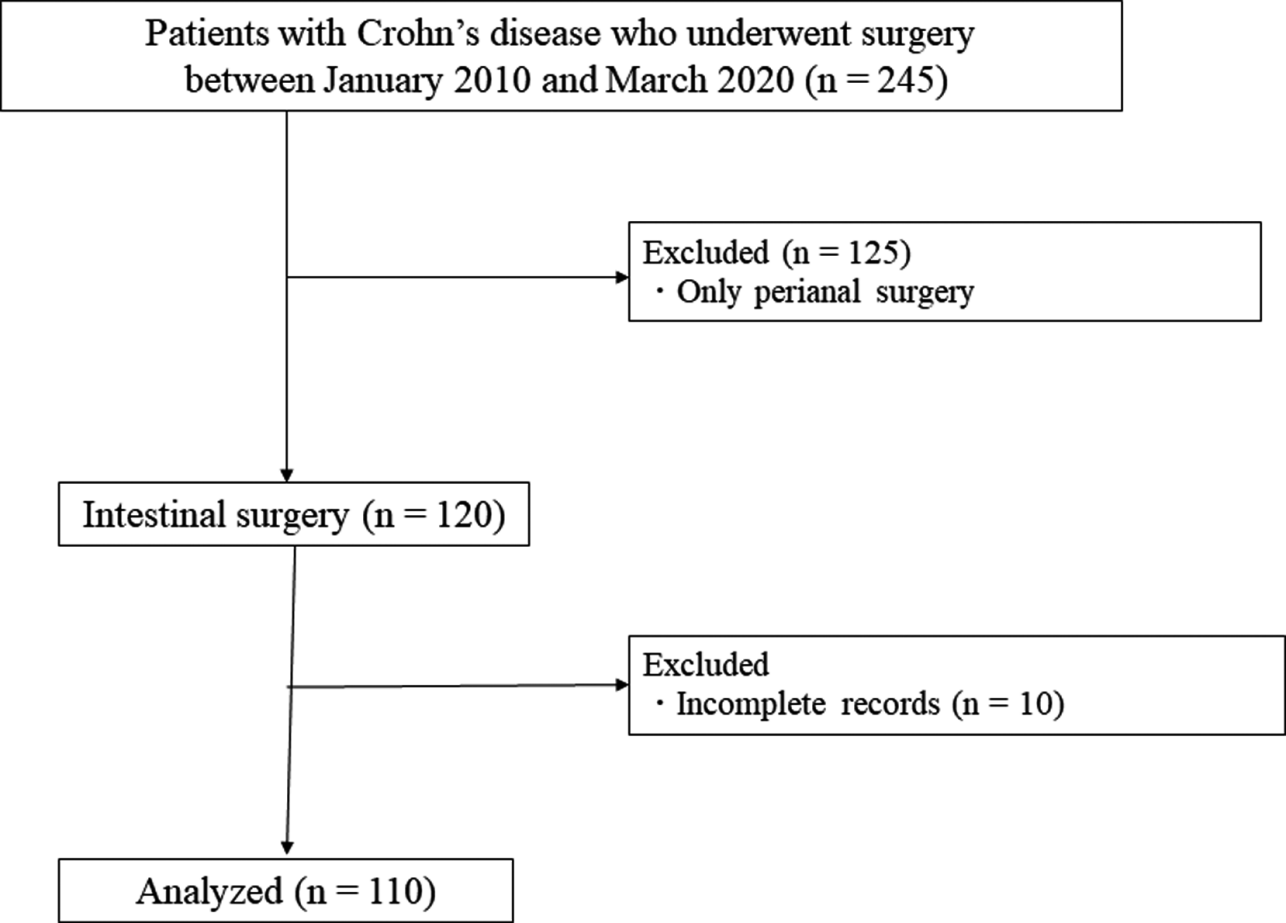
Flow chart of participant inclusion in the study

A total of 245 consecutive patients who underwent CD surgery between January 2010 and March 2020 at Osaka University Hospital were included in this study. We excluded 125 patients who underwent perianal surgery only and 10 patients with incomplete records (Figure 1). Informed consent was obtained from all patients before the surgery. The study protocol was approved by the Institutional Review Board of Osaka University Hospital (# 15028).

### 2.2 Perioperative management and surgical procedure

Prophylactic antibiotics with second‐generation cephalosporin (cefmetazole) were intravenously administered within 30 min before skin incision, repeatedly at 3‐h intervals during surgery, and postoperatively twice a day until postoperative day (POD) 2. Drainage tubes were placed in a Morrison's pouch or Douglas’ pouch. Before March 2012, we performed closed passive drainage using surgeon's choice of a SILASCON duple drain (Kaneka Medical Products, Japan) or closed active drainage using a BLAKE silicone drain (Ethicon, Cincinnati, OH). After April 2012, closed active drainage was used in all cases.

A wound protector was used during surgery, and intraperitoneal lavage was performed before wound closure. All surgical staff changed gloves after bowel anastomosis. The peritoneum was closed by a running suture with 3‐0 Vicryl (Ethicon). The muscular fascia was closed by a knotted suture with 1‐PDS PLUS (Ethicon). The wound was washed with 200 mL of saline before closure of the skin by a buried suture with 4‐0 PDS plus (Ethicon). Board‐certified surgeons performed all surgeries.

### 2.3 Outcome measurement and definition of SSI

For evaluating candidate risk factors for SSI, we collected medical records data on patient characteristics including age, sex, body mass index (BMI), American Society of Anesthesiologists score, duration of disease, behavior (nonstricturing and nonpenetrating, stricturing, or penetrating), preoperative laboratory data, antitumor necrosis factor alpha (TNF)‐α use, steroid use, and frequency of surgery. We also collected data on surgical variables, including type of surgery (open or laparoscopic), surgical site (small bowel, colon, ileocolon, or rectum), with or without abdominoperineal resection (APR), ostomy creation, surgical wound classification, duration of surgery, intraoperative bleeding, transfusion, and D‐Posi. The surgical staff examined the wounds daily, and bacterial culture of drainage fluid was performed on POD 1 and 4 in all cases. The definition of D‐Posi was detection of bacteria in the drainage fluid at POD 1 or 4.

Surgical site infection was defined according to the US Centers for Disease Control and Prevention classifications,^4^, ^15^ as an infection that occurred at the surgical site within 30 d after surgery with at least one of the following observations: purulent discharge from the incision or from the drain placed into the organ/space; bacteria isolated by culturing fluid or tissue from the incision or the organ/space; an open wound with signs and symptoms of infection; and/or an abscess or other evidence of infection found on examination of the incision or the organ/space.^11^, ^16^

### 2.4 Detection method of bacterial species

Bacterial culture of drainage fluid was performed on the same day a sample was submitted. The sample was smeared for detecting the type by gram staining, and then coated on both Agar’s and Ringer’s medium. Results of the gram staining were available the same day. The bacterial species were examined with matrix‐assisted laser desorption/ionization‐time‐of‐flight mass spectrometry using colonies on Agar medium. On the day following sample submission, D‐Posi status and bacterial species were identified. The antibiotic sensitivity was identified at 2 d after sample submission. If the amount of bacteria was limited, this step sometimes took a few more days and up to 1–2 wk for very low counts associated with using Ringer’s medium.

### 2.5 Statistical analysis

All statistical analyses were performed using JMP 15 (SAS Institute, Cary, NC).

The reference values were determined according to the median values about age, duration of disease, C‐reactive protein, duration of surgery, and intraoperative bleeding, and also according to the standard values of white blood cell (WBC), hemoglobin, platelet and albumin, and BMI. Patients’ backgrounds, surgical variables, and postoperative results were compared using the Chi‐squared or Fisher's exact test for categorical variables, as appropriate. Odds ratios (ORs) were used to evaluate the relationship between SSI and different factors. Variables from comparisons yielding *P* < .1 in the univariate analysis were entered into a multivariate analysis. For multivariate analysis, we used a logistic regression model to investigate the factors associated with SSI incidence. The predictive accuracy and area under the curve (AUC) were calculated. Receiver operating characteristic analyses were performed to compare the performance of variables as SSI risk predictors. Risk factors, excluding D‐Posi, with a univariate *P* < .1, were defined as “Others” and “Others” together with D‐Posi was defined as “Combination.” Diagnostic accuracy was judged using the diagnostic OR (DOR), which is not affected by disease frequency and incidence of complications. Statistical significance was defined as *P* < .05. The ratios were calculated as follows:

Positive likelihood ratio (LR (+))=sensitivity/(1‐specificity);

Negative likelihood ratio (LR (‐))=(1‐sensitivity)/specificity;

DOR=positive LR/negative LR.

## 3 RESULTS

### 3.1 Patient characteristics

Data for 110 patients were analyzed and the characteristics of the 84 men and 26 women are shown in Table 1. The median age at surgery was 39.5 y, and 36 patients (32.7%) were D‐Posi. The APR accounted for 10 patients (9.1%). The median duration of surgery was 203.5 min, and the median intraoperative bleeding was 145 mL. Seven patients (6.4%) received a blood transfusion. A total of 17 patients (15.5%) received preoperative antibiotics for high inflammatory response with penetrating disease.

**TABLE 1:** Patient characteristics (N = 110)

	N*
Preoperative	Median (range)
Age, y	39.5 (14–72)
Male/female	84/26
BMI, kg/m^2^	19.0 (14.0–28.4)
ASA score ≥2, n (%)	71 (64.5%)
Duration of disease, y	10 (0–41)
Behavior	
Nonstricturing & nonpenetrating	7 (6.4%)
Stricturing	41 (37.2%)
Penetrating	62 (56.4%)
Preoperative WBC, per µL	5260 (1990–16 890)
Preoperative Hb, g/dL	12.0 (7.3–16.1)
Preoperative platelet ×10^4^, per µL	28.8 (6.8–57.8)
Preoperative albumin, g/dL	3.5 (1.5–4.9)
Preoperative CRP, mg/dL	0.20 (0–22.4)
Preoperative use of anti‐TNF‐α antibody	78 (70.9%)
Preoperative use of steroid	11 (10%)
Frequency of surgery (primary/redo)	66/44
Surgical	
Open/laparoscopic	13/97
Surgical site	
Small bowel/colon/ileocolon/rectum	21/14/65/10
APR, n (%)	10 (9.1%)
Ostomy creation	18
Wound class (II/III/IV)	95/8/7
Duration of surgery, min	203.5 (105–748)
Intraoperative bleeding, mL	145 (0–8050)
Transfusion, n (%)	7 (6.4%)
D‐Posi, n (%)	36 (32.7%)

Abbreviations: APR, abdominoperineal resection; ASA, American Society of Anesthesiologists; BMI, body mass index; CRP, C‐reactive protein; D‐Posi, bacterial culture positive of postoperative drainage fluid; Hb, hemoglobin; WBC, white blood cell count.

* Median (range) unless otherwise specified.

### 3.2 Postoperative complications

Postoperative complications were diagnosed in 28 patients (25.5%), including overall SSI in 18 (16.4%), incisional SSI in 16 (14.5%), and organ/space SSI in six (5.5%) (12 had incisional SSI only, two had organ/space SSI only, and four had both types of SSI). Gastrointestinal bleeding occurred in five patients (4.5%), intra‐abdominal bleeding in one (0.9%), subcutaneous bleeding in one (0.9%), ileus in five (4.5%), catheter infection in four (3.6%), and femoral nerve palsy in one (0.9%). Severe complications (Clavien–Dindo grade ≥ III) were observed in seven patients (6.4%). There were no perioperative deaths.

### 3.3 SSI risk factors

For analyses, 18 patients were in the overall SSI group and compared with the other 92 patients in the nonoverall SSI group. Univariate analysis of risk factors associated with overall SSI identified preoperative WBC ≥9400/µL, open surgery, APR, a longer duration of surgery, a high degree of intraoperative bleeding, and D‐Posi (Table 2). Risk factors for incisional SSI were WBC ≥9400/µL, APR, a longer duration of surgery, and D‐Posi; the only risk factor associated with organ/space SSI was D‐Posi. Of the patients with incisional SSI, 68.8% (11/16) were D‐Posi, whereas 26.6% (25/ 94) without incisional SSI were D‐Posi (*P* = .0021). Similarly, 83.3% (5/6) with organ/space SSI were D‐Posi, and 29.8% (31/104) without organ/space were D‐Posi (*P* = .027) (Table 3). In multivariate analyses, only D‐Posi emerged as an independent risk factor of overall, incisional, and organ/space SSI. In addition, high BMI was also an independent risk factor for incisional SSI (Table 4).

**TABLE 2:** Univariate analysis of risk factors associated with overall surgical site infection (SSI)

	Overall SSI group, n (%) (n = 18)	Nonoverall SSI group, n (%) (n = 92)	OR	95% CI	*P*
Preoperative backgrounds					
Age ≥40 y	10 (55.6%)	45 (48.9%)	1.31	0.47–3.60	.607
Male	15 (83.3%)	69 (75.0%)	1.67	0.44–6.28	.450
BMI ≥22 kg/m^2^	7 (38.9%)	21 (22.8%)	2.15	0.74–6.24	.159
ASA score ≥2	12 (66.7%)	59 (64.1%)	1.12	0.38–3.26	.837
Duration of disease ≥10 y	9 (50.0%)	47 (51.6%)	0.94	0.34–2.57	.898
Behavior					
Penetrating/nonpenetrating	8/10	54/38	0.56	0.20–1.56	.269
Preoperative WBC ≥9.4 × 10^3^/µL	5 (27.8%)	9 (9.8%)	3.55	1.03–12.3	.045^a^
Preoperative Hb <12 g/dL	12 (66.7%)	43 (46.7%)	2.28	0.79–6.59	.129
Preoperative platelet ≥32 × 10^4^/µL	9 (50.0%)	31 (33.7%)	1.97	0.71–5.46	.193
Preoperative albumin <3.5 g/dL	9 (52.9%)	38 (41.8%)	1.57	0.55–4.44	.396
Preoperative CRP ≥0.2 mg/dL	11 (61.1%)	43 (46.7%)	1.79	0.64–5.03	.269
Preoperative use of anti‐TNF‐α antibody	13 (72.2%)	65 (70.7%)	1.08	0.35–3.33	.893
Preoperative use of steroid	2 (11.1%)	9 (9.8%)	1.15	0.22–5.84	.864
Frequency of surgery; redo	9 (50.0%)	35 (38.0%)	1.63	0.59–4.50	.347
Surgical backgrounds					
Open/Laparoscopic	5/13	8/84	4.04	1.14–14.2	.030^a^
Surgical site					
Small bowel	3	18	0.25	0.04–1.45	.123
Colon	2	12	0.25	0.04–1.77	.166
Ileocolon	9	56	0.24	0.06–1.03	.054
Rectum	4	6	Reference = 1		
APR	4 (22.2%)	6 (6.52%)	4.10	1.02–16.4	.046^a^
Ostomy creation	5 (27.8%)	13 (14.1%)	2.34	0.71–7.66	.161
Wound class ≥III	3 (16.7%)	12 (13.0%)	1.33	0.34–5.30	.683
Duration of surgery ≥204 min	14 (77.8%)	41 (44.6%)	4.35	1.33–14.2	.015^a^
Intraoperative bleeding ≥145 mL	13 (72.2%)	42 (45.7%)	3.10	1.02–9.39	.046^a^
Transfusion	2 (11.1%)	5 (5.4%)	2.18	0.39–12.2	.377
D‐Posi	13 (72.2%)	23 (25.0%)	7.80	2.51–24.2	.0004^a^

Abbreviations: 95% CI, 95% confidence interval; APR, abdominoperineal resection; ASA, American Society of Anesthesiologists; BMI, body mass index; CRP, C‐reactive protein; D‐Posi, bacterial culture–positive postoperative drainage fluid; Hb, hemoglobin; OR, odds ratio; WBC, white blood cell count.

^a^ *P* < .05

**TABLE 3:** Univariate analysis of risk factors associated with incisional surgical site infection (SSI) and organ space SSI

	Incisional SSI				Organ/space SSI			
	Incisional SSI, n (%) (n = 16)	Nonincisional SSI, n (%) (n = 94)	OR (95% CI)	*P*	Organ/space SSI, n (%) (n = 6)	Nonorgan/space SSI, n (%) (n = 104)	OR (95% CI)	*P*
Preoperative backgrounds								
Age ≥40 y	10 (62.5%)	45 (47.9%)	1.8 (0.61–5.40)	.284	1 (16.7%)	54 (51.9%)	0.19 (0.02–1.64)	.130
Male	14 (87.5%)	70 (74.5%)	2.4 (0.51–11.34)	.269	4 (66.7%)	80 (76.9%)	0.60 (0.10–3.48)	.569
BMI ≥22 kg/m^2^	7 (43.8%)	21 (22.3%)	2.7 (0.90–8.13)	.077	1 (16.7%)	27 (26.0%)	0.57 (0.06–5.10)	.616
ASA score ≥2	10 (62.5%)	61 (64.9%)	0.90 (0.30–2.70)	.853	4 (66.7%)	67 (64.4%)	1.1 (0.19–6.32)	.911
Duration of disease ≥10 y	8 (50.0%)	48 (51.6%)	0.94 (0.32–2.71)	.905	4 (66.7%)	52 (50.5%)	2.0 (0.34–11.18)	.448
Behavior								
Penetrating/ nonpenetrating	6/10	56/38	0.41 (0.14–1.21)	.11	4/2	58/48	1.59 (0.28–9.05)	.604
Preoperative WBC ≥9.4 × 10^3^/µL	5 (31.3%)	9 (9.6%)	4.3 (1.22–15.15)	.024^a^	1 (16.7%)	13 (12.5%)	1.4 (0.15–12.95)	.767
Preoperative Hb <12 g/dL	10 (62.5%)	45 (47.9%)	1.8 (0.61–5.40)	.284	4 (66.7%)	51 (49.0%)	2.1 (0.36–11.85)	0.410
Preoperative platelet ≥32 × 10^4^/µL	9 (56.3%)	31 (33.0%)	2.6 (0.89–7.67)	.081	2 (33.3%)	38 (36.5%)	0.87 (0.15–4.97)	.874
Preoperative albumin <3.5 g/dL	7 (46.7%)	40 (43.0%)	1.2 (0.39–3.46)	.791	3 (50.0%)	44 (43.1%)	1.3 (0.25–6.85)	.742
Preoperative CRP ≥0.2 mg/dL	11 (68.8%)	43 (45.7%)	2.6 (0.84–8.10)	.097	2 (33.3%)	52 (50.0%)	0.50 (0.09–2.85)	.435
Preoperative use of anti‐TNF‐α antibody	11 (68.8%)	67 (71.3%)	0.89 (0.28–2.79)	.837	6 (100%)	72 (69.2%)	NA	NA
Preoperative use of steroid	2 (12.5%)	9 (9.6%)	1.35 (0.26–6.91)	.719	1 (16.7%)	10 (9.6%)	1.90 (0.20–17.73)	.581
Frequency of surgery; redo	8 (50.0%)	36 (38.3%)	1.61 (0.56–4.67)	.380	3 (50.0%)	41 (39.4%)	1.54 (0.30–7.98)	.609
Surgical backgrounds								
Open/laparoscopic	4/12	9/85	3.1 (0.84–11.83)	.090	2/4	11/93	4.2 (0.69–25.8)	.118
Surgical site								
Small bowel/ Colon or ileocolon/ rectum	2 10 4	19 69 6	0.16 (0.02–1.09) 0.22 (0.05–0.91) Reference=1	.061 .036^a^	2 2 2	19 77 8	0.42(0.05–3.53) 0.10(0.01–0.84) Reference=1	.425 .034^a^
APR	4 (25.0%)	6 (6.4%)	4.9 (1.20–19.86)	.027^a^	2 (33.3%)	8 (7.7%)	6.0 (0.95–37.94)	.057
Ostomy creation	4 (25.0%)	14 (25.9%)	1.9 (0.53–6.76)	.319	2 (33.3%)	16 (15.4%)	2.8 (0.46–16.29)	.265
Wound class ≥III	2 (12.5%)	13 (13.8%)	0.89 (0.18–4.38)	.886	1 (16.7%)	14 (13.5%)	1.29 (0.140–11.83)	.824
Duration of surgery ≥204 min	12 (75.0%)	43 (45.7%)	3.6 (1.07–11.84)	.039^a^	5 (83.3%)	50 (48.1%)	5.4 (0.61–47.83)	.130
Intraoperative bleeding ≥145 mL	11 (68.8%)	44 (46.8%)	2.5 (0.81–7.76)	.113	5 (83.3%)	50 (48.1%)	5.4 (0.61–47.83)	.130
Transfusion	2 (12.5%)	5 (5.3%)	2.5 (0.45–14.4)	.292	0 (0%)	7 (6.7%)	NA	NA
D‐Posi	11 (68.8%)	25 (26.6%)	6.1 (1.92–19.21)	.0021^a^	5 (83.3%)	31 (29.8%)	11.8 (1.32–105.0)	.027^a^

Abbreviations: 95% CI, 95% confidence interval; APR, abdominoperineal resection; ASA, American Society of Anesthesiologists; BMI, body mass index; CRP, C‐reactive protein; D‐Posi, bacterial culture–positive postoperative drainage fluid; Hb, hemoglobin; NA, not applicable; OR, odds ratio; WBC, white blood cell count.

^a^ *P* < .05.

**TABLE 4:** Multivariate analysis of risk factors associated with surgical site infection (SSI)

	OR	95% CI	*P*
Overall SSI			
Preoperative WBC ≥9.4 × 10^3^/µL	1.83	0.40–8.44	.439
Open	1.59	0.37–6.92	.534
APR	1.82	0.32–10.3	.495
Duration of surgery ≥204 min	2.49	0.55–11.3	.237
Intraoperative bleeding ≥145 mL	1.30	0.31–5.48	.722
D‐Posi	6.00	1.79–19.9	.0036^a^
Incisional SSI			
BMI ≥22 kg/m^2^	4.77	1.17–19.5	.030^a^
Preoperative WBC ≥9.4 × 10^3^/µL	1.60	0.28–9.07	.598
Preoperative platelet ≥32 × 10^4^/µL	2.90	0.71–11.9	.139
Preoperative CRP ≥0.2 mg/dL	1.52	0.32–7.23	.598
Open	2.04	0.38–11.1	.408
APR	2.91	0.43–19.9	.276
Duration of surgery ≥204 min	1.78	0.39–8.13	.451
D‐Posi	5.59	1.42–22.0	.014^a^
Organ/space SSI			
APR	4.63	0.65–33.0	.126
D‐Posi	10.6	1.26–96.1	.037^a^

Abbreviations: 95% CI, 95% confidence interval; APR, abdominoperineal resection; D‐Posi, bacterial culture–positive postoperative drainage fluid; OR, odds ratio; WBC, whiteblood cell count.

^a^ *P* < .05.

Abdominoperineal resection has been reported as a risk factor for incisional and organ/space SSI, leading to assess also the clinical impact of D‐Posi with exclusion of APR.^11^ Of 100 patients with APR excluded, overall SSI developed in 14 patients: 10 with incisional SSI only, two with organ/space only, and two with both. Eleven (78.6%) with overall SSI were D‐Posi, whereas 20 (23.3%) without overall SSI were D‐Posi (*P* = .0004). We found that 75% with incisional SSI were D‐Posi and 22.0% without incisional SSI were D‐Posi (*P* = .002). Similarly, 75% with organ/space SSI were D‐Posi, and 29.2% without organ/space SSI were D‐Posi (*P* = .091). Multivariate analysis identified D‐Posi as an independent risk factor in overall SSI and incisional SSI also among the patients with APR excluded.

### 3.4 Treatment details for the case of D‐Posi

Of 110 patients, 36 were D‐Posi, 19 (52.8%) of whom received only prophylactic antibiotics. Four (11.1%) had longer administration of cefmetazole for a few days. Nine (25.0%) were switched to other antibiotics with broad‐spectrum activity, based on the detected bacterial species. Four (11.1%) were switched to other antibiotics and had percutaneous drainages. Among the patients with D‐Posi, confirming their status took a median of 2 d in 13 patients with SSI and 5 d in 23 patients with non‐SSI. Identifying antibiotic sensitivity took a median of 4 (2–17) d. The period for detecting D‐Posi was significantly shorter in the SSI group compared to the non‐SSI group (*P* = .007), possibly because of different bacterial loads.

### 3.5 Comparison of predictive and diagnostic accuracy

**FIGURE 2:**
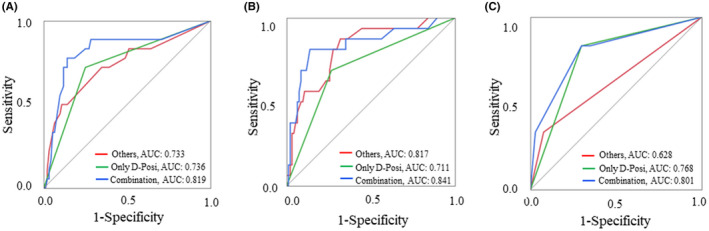
Receiver operating characteristic curve analysis based on risk factors other than D‐Posi, D‐Posi, or a combination of both for surgical site infection (SSI). (A) Overall SSI. (B) Incisional SSI. (C) Organ/space SSI. AUC, area under the curve. D‐Posi, positive bacterial culture of drainage fluid

Next, we evaluated the predictive and diagnostic ability of D‐Posi. In overall SSI, the AUC for D‐Posi was 0.736, and the DOR was 7.81, and the AUC was higher than the “Others” group (AUC = 0.733). Values for the “Combination” were the highest, with an AUC of 0.819 and DOR of 21.3 (Figure 2A and Table 5). In organ/space SSI, the AUC for D‐Posi was 0.768, and the DOR was 11.8 (Figure 2C and Table 5), while D‐Posi was not a strong predictor for incisional SSI compared to “Others” and “Combination,” with an AUC of 0.711 and DOR of 6.07 (Figure 2B and Table 5). The negative predictive value was above 90% in all groups.

**TABLE 5:** Diagnostic accuracies for predicting surgical site infection (SSI)

	Sensitivity (%)	Specificity (%)	PPV (%)	NPV (%)	LR (+)	LR (−)	DOR
Overall SSI							
Others	50.0	89.1	47.4	90.1	4.60	0.56	8.20
D‐Posi	72.2	75.0	36.1	93.2	2.89	0.37	7.81
Combination	77.8	85.9	51.9	95.2	5.50	0.26	21.3
Incisional SSI							
Others	87.5	68.1	31.8	97.0	2.74	0.18	14.9
D‐Posi	68.8	73.4	30.6	93.2	2.59	0.43	6.07
Combination	81.3	86.2	50.0	96.4	5.88	0.22	27.0
Organ/space SSI							
Others	33.3	92.3	20.0	96.0	4.33	0.72	6.00
D‐Posi	83.3	70.2	13.9	98.6	2.80	0.24	11.8
Combination	83.3	70.2	13.9	98.6	2.80	0.24	11.8

Abbreviations: DOR, diagnostic odds ratio; D‐Posi, bacterial culture–positive postoperative drainage fluid; LR (−), negative likelihood ratio; LR (+), positive likelihood ratio; NPV, negative predictive value; PPV, positive predictive value.

^a^ *P* < .05.

### 3.6 Characteristics of detected bacteria

We investigated which bacteria were associated with SSI risk. In univariate analysis, *Pseudomonas aeruginosa* and *Enterococcus faecalis* were significantly associated with overall SSI (*P* = .0024 and .017, respectively). *Pseudomonas aeruginosa* was frequently detected in patients with incisional and organ/space SSI *Klebsiella pneumoniae* and *E. faecalis* were often frequently detected in patients with organ/space SSI (Table S1). The comparison of detected bacteria between SSI and D‐Posi were also performed. Of 16 patients with incisional SSI, seven (43.8%) had the same organism detected from both samples. Among six patients with organ/space SSI, four (66.6%) had the same organism in both samples (Table S2). Of 18 patients with SSI, six organisms were subjected to an antibiotic sensitivity test of both samples. Most of the antibiotic sensitivity results were the same in both samples, and the examined organisms were resistant to the prophylactic antibiotic cefmetazole (Table S3).

Of 110 patients, only four patients underwent bacterial culture of intraoperative samples (*Proteus vulgaris* detected in one case, *Bacteroides fragilis* in one, *K. pneumoniae* and *Escherichia coli* in one, and none detected in the fourth case). Of these four patients, two were D‐Posi, but the detected bacteria were different from the intraoperative samples, and none of the four patients developed SSI.

## 4 DISCUSSION

In patients with CD, it is important to detect SSI in the early phase and prevent worsening, because infectious complications could increase the recurrence risk.^7^, ^8^, ^17^, ^18^ Thus, accurate predictors of SSI are needed in this patient population. Several studies have suggested that D‐Posi could have predictive associations with predicting SSI.^12^, ^14^ Sugiura et al reported that bacterial culture‐positive peritoneal lavage fluid correlated with SSI and pancreatic fistula after pancreaticoduodenectomy.^12^ Migita et al showed that D‐Posi was an independent risk factor among clinical and surgical variables for intra‐abdominal abscess after gastrectomy.^14^ Routine use of prophylactic drainage has been reported not to decrease postoperative complications in colorectal surgeries, including ileocecal resection or right hemicolectomy.^19^, ^20^ However, undrained fluid could cause intra‐abdominal abscess because of bacterial leakage from the mesentery in patients with CD.^21^ Compared with patients with cancer, patients with CD also have a higher risk of infection because of low nutrition, the use of immunosuppressive drugs, and the presence of fistulas and abscess. Therefore, we routinely placed prophylactic drainage for our patients with CD. This study is the first to investigate the impact of D‐Posi and detected bacteria in these patients. The results identified D‐Posi as an independent predictor of SSI, with an AUC >0.8 in the early phase.

Organ/space SSI usually becomes evident around POD 7 or later.^22^, ^23^ To prevent worsening, early administration of antibiotics is recommended for suspected cases after gastrointestinal surgery.^24^, ^25^ Percutaneous drainage also can preclude the need for surgical intervention for intra‐abdominal abscess.^26^ In our study, six patients had organ/space SSI, and all were diagnosed at POD 8 (POD 5–15) because computed tomography diagnosis of organ/space SSI was mainly performed following symptom progression. The negative predictive value of diagnosing SSI was >95% with the use of D‐Posi, suggesting its potential to allow prediction of SSI on POD 4. Of six patients with organ/space SSI, four with D‐Posi were switched to other antibiotics with broad‐spectrum activity on POD 4.5 (POD 2–7), and one patient continued receiving antibiotics from 14 d before surgery until POD 11 because of high inflammation with penetrating disease. Identifying the D‐Posi status might allow us perform empirical treatment in the early phase before the definitive diagnosis.

Several studies have demonstrated that patients with CD have gut bacterial alterations characterized by reduced bacterial diversity, depletion of Firmicutes or Bacteroides, and enrichment of Proteobacteria.^1^, ^2^, ^27^, ^28^, ^29^ However, the causative bacteria of SSI in patients with CD have not been reported. A previous study showed that *E. faecalis*, *S. aureus,* and *P. aeruginosa* were often isolated from SSI following lower intestinal tract surgery.^30^ Our data also showed that *E. faecalis* and *P. aeruginosa* were significantly detected in CD patients with organ/space SSI. Because *P.aeruginosa* affected immunosuppressed patients, careful management would be essential for CD patients under treatment.^31^, ^32^ The current findings suggest that organisms at the site of SSI were resistant to the prophylactic antibiotic cefmetazole and that most of the antibiotic sensitivity results were the same between drainage fluid and of SSI bacterial culture. This suggested that sensitivity results in drainage fluid could guide the choice of an antibiotic.

The present study had several limitations. First, these results were based on a single‐center retrospective cohort study, including some selection bias that could not be avoided. Second, the impact of preoperative medicines, such as biologics except anti‐TNF‐α therapy, immune suppressants, and 5‐aminosalicylic acid were not evaluated in this study. A larger study that includes these factors is needed.

In conclusion, this study confirmed the clinical usefulness of D‐Posi for SSI and demonstrated the association of bacteria species with CD surgery. Bacterial culture of drainage fluid represents an easy, noninvasive, and inexpensive tool to perform appropriate management of SSI.

## DISCLOSURES

The study protocol was approved by the Institutional Review Board of Osaka University Hospital (# 15028).

Informed consent was obtained from all patients before the surgery.

This research was not preregistered in an independent, institutional registry (N/A).

This research was not an animal study (N/A).

The authors T.O. and T.M. were supported by the KINSHUKAI GROUP.

Author T.M. was partly supported by a research grant from the Osaka Medical Research Foundation for Intractable Disease, JSP KAKENHI (20K17615), Danone Institute of Japan Foundation (DIJF R01‐24), and Astellas Pharma Global Development (Grant No. IDRS2017A0000577).

## AUTHOR CONTRIBUTIONS

Study concept and design: M.I., T.O., and T.M. Acquisition of data: M.I., T.O., and T.M. Statistical analysis and interpretation of data: M.I., T.O., and M.F. Drafting of the article: M.I. and T.O. Critical revision of the article for important intellectual content: all authors. Supervision: Y.D. and H.E.

## Supporting information

Table S1‐S3
