# Supplementary material for: Usefulness of Bacterial Culture of Drainage Fluid for Predicting Surgical Site Infection After Crohn’s Disease Surgery
Source: Ann Gastroenterol Surg. 2021 Nov 27;6(3):375–85. doi: 10.1002/ags3.12530 (PMC9130903; doi:10.1002/ags3.12530)
Supplement: Supplementary file 1 — Table S1‐S3 [file AGS3-6-375-s001.docx]

Supplemental table 1. Association between SSI and the detected bacteria

|  | **Overall SSI** | | |  | **Incisional SSI** | | |  | **Organ/space SSI** | | |
| --- | --- | --- | --- | --- | --- | --- | --- | --- | --- | --- | --- |
|  | **Overall**  **SSI**  **(n = 18)** | **Non-**  **overall**  **SSI**  **(n = 92)** | ***P*** |  | **Incisional**  **SSI**  **(n = 16)** | **Non-**  **Incisional**  **SSI**  **(n = 94)** | ***P*** |  | **Organ**  **/space**  **SSI**  **(n = 6)** | **Non-**  **Organ**  **/space**  **SSI**  **(n = 104)** | ***P*** |
| Gram-negative rod |  |  |  |  |  |  |  |  |  |  |  |
| *Escherichia coli* | 1 (5.6%) | 5 (5.4%) | 1.000 |  | 1 (6.3%) | 5 (5.3%) | 1.000 |  | 0 (0%) | 6 (5.8%) | 1.000 |
| *Klebsiella pneumoniae* | 2 (11.1%) | 1 (1.1%) | 0.069 |  | 1 (6.3%) | 2 (2.1%) | 0.379 |  | 2 (33.3%) | 1 (0.96%) | 0.0073^†^ |
| *Pseudomonas aeruginosa* | 4 (22.2%) | 1 (1.1%) | 0.0024^†^ |  | 3 (18.8%) | 2 (2.1%) | 0.021^†^ |  | 3 (50%) | 2 (1.9%) | 0.0009^†^ |
| *Citrobacter freundii* | 1 (5.6%) | 0 (0%) | 0.164 |  | 1 (6.3%) | 0 (0%) | 0.146 |  | 0 (0%) | 1 (0.96%) | 1.000 |
| *Enterobacter sp.* | 2 (11.1%) | 1 (1.1%) | 0.069 |  | 2 (12.5%) | 1 (1.1%) | 0.055 |  | 1 (16.7%) | 2 (1.9%) | 0.156 |
| *Bacteroides fragilis group* | 4 (22.2%) | 6 (6.5%) | 0.057 |  | 3 (18.8%) | 7 (7.5%) | 0.160 |  | 1 (16.7%) | 9 (8.7%) | 0.443 |
| Gram-positive rod |  |  |  |  |  |  |  |  |  |  |  |
| *Staphylococcus epidermidis* | 0 (0%) | 2 (2.2%) | 1.000 |  | 0 (0%) | 2 (2.1%) | 1.000 |  | 0 (0%) | 2 (1.9%) | 1.000 |
| *Lactobacillus sp.* | 0 (0%) | 1 (1.1%) | 1.000 |  | 0 (0%) | 1 (1.1%) | 1.000 |  | 0 (0%) | 1 (0.96%) | 1.000 |
| *Clostridium sp.* | 2 (11.1%) | 1 (1.1%) | 0.069 |  | 1 (6.3%) | 2 (2.1%) | 0.379 |  | 1 (16.7%) | 2 (1.9%) | 0.156 |
| *Bifidobacterium sp.* | 0 (0%) | 1 (1.1%) | 1.000 |  | 0 (0%) | 1 (1.1%) | 1.000 |  | 0 (0%) | 1 (0.96%) | 1.000 |
| *Bacillus sp.* | 0 (0%) | 1 (1.1%) | 1.000 |  | 0 (0%) | 1 (1.1%) | 1.000 |  | 0 (0%) | 1 (0.96%) | 1.000 |
| Gram-positive cocci |  |  |  |  |  |  |  |  |  |  |  |
| *Micrococcus sp.* | 1 (5.6%) | 0 (0%) | 0.164 |  | 0 (0%) | 1 (1.1%) | 1.000 |  | 1 (16.7%) | 0 (0%) | 0.055 |
| *Enterococcus faecalis* | 5 (27.8%) | 6 (6.5%) | 0.017^†^ |  | 3 (18.8%) | 8 (8.5%) | 0.199 |  | 4 (66.7%) | 7 (6.7%) | 0.0008^†^ |
| Fungus (*Candida*) | 0 (0%) | 2 (2.2%) | 1.000 |  | 0 (0%) | 2 (2.1%) | 1.000 |  | 0 (0%) | 2 (1.9%) | 1.000 |
| Total | 22 | 28 |  |  | 15 | 35 |  |  | 13 | 37 |  |

^†^ *P* < 0.05

Supplemental table 2. Results for bacterial culture of SSI versus drainage fluid

| Patient No. | **Bacterial culture of**  **drainage fluid** | **Incisional SSI** | **Bacterial culture of**  **Incisional**  **SSI** | **Organ/space**  **SSI** | **Bacterial culture of**  **Organ/space**  **SSI** |
| --- | --- | --- | --- | --- | --- |
| Patient 1 | ***Klebsiella pneumoniae /*** *Pseudomonas aeruginosa* | **(+)** | *Proteus sp.* | **(+)** | ***Klebsiella pneumoniae*** */ Enterococcus faecalis* ***/*** *Proteus sp.* |
| Patient 2 | ***Enterococcus faecalis / Enterobacter sp.*** | **(+)** | ***Enterococcus faecalis / Enterobacter sp.*** | **(+)** | ***Enterococcus faecalis / Enterobacter sp.*** |
| Patient 3 | ***Pseudomonas aeruginosa*** */ Enterococcus faecalis* | **(+)** | Not examined | **(+)** | ***Pseudomonas aeruginosa*** */ Enterococcus avium* |
| Patient 4 | Negative | **(+)** | Enterococcus faecalis | **(+)** | *Enterococcus faecalis* |
| Patient 5 | *Bacteroides fragilis group* | **(+)** | Not examined | **(-)** |  |
| Patient 6 | ***Clostridium sp.*** | **(+)** | ***Clostridium sp.*** */ Bacteroides sp.* | **(-)** |  |
| Patient 7 | *Bacteroides fragilis group* | **(+)** | *Enterococcus raffinosus / Klebsiella pneumoniae* | **(-)** |  |
| Patient 8 | ***Citrobacter freundii*** | **(+)** | ***Citrobacter freundii*** */ Enterococcus faecalis* | **(-)** |  |
| Patient 9 | ***Pseudomonas aeruginosa*** | **(+)** | ***Pseudomonas aeruginosa*** */ Staphylococcus epidermidis* | **(-)** |  |
| Patient 10 | ***Enterococcus faecalis*** | **(+)** | ***Enterococcus faecalis*** | **(-)** |  |
| Patient 11 | ***Escherichia coli*** */ Bacteroides fragilis group* | **(+)** | ***Escherichia coli*** | **(-)** |  |
| Patient 12 | ***Enterobacter sp.*** | **(+)** | ***Enterobacter sp.*** */ Enterococcus faecalis* | **(-)** |  |
| Patient 13 | Negative | **(+)** | *Anaero-gram-rod+* | **(-)** |  |
| Patient 14 | Negative | **(+)** | *Enterococcus faecalis* | **(-)** |  |
| Patient 15 | Negative | **(+)** | *Enterococcus faecium* | **(-)** |  |
| Patient 16 | Negative | **(+)** | Not examined | **(-)** |  |
| Patient 17 | *Bacteroides fragilis group / Enterococcus faecalis / Clostridium sp. / Micrococcus sp.* | **(-)** |  | **(+)** | Not examined |
| Patient 18 | ***Pseudomonas aeruginosa*** */ Enterococcus faecalis / Klebsiella pneumoniae* | **(-)** |  | **(+)** | ***Pseudomonas aeruginosa*** |

Bold indicated the same bacteria detected.

Supplemental table 3. Antibiotic sensitivity between drainage fluid and bacterial culture of SSI

|  | **Patient number and detected bacteria** | | | | | | | | | | | | | | | | | | | | | |  |  |
| --- | --- | --- | --- | --- | --- | --- | --- | --- | --- | --- | --- | --- | --- | --- | --- | --- | --- | --- | --- | --- | --- | --- | --- | --- |
|  | **3**  ***Pseudomonas Aeruginosa*** | |  |  | **8**  ***Citrobacter freudii*** | | | |  | **9**  ***Pseudomonas***  ***aeruginosa*** | |  | **10**  ***Enterococcus faecalis*** | |  | **12**  ***Enterobacter***  ***sp.*** | |  | **18**  ***Pseudomonas aeruginosa*** | | | |  |  |
| **Antibiotics** | **Drainage**  **fluid** | **SSI** |  | |  | **Drainage**  **fluid** | | **SSI** |  | **Drainage**  **fluid** | **SSI** |  | **Drainage**  **fluid** | **SSI** |  | **Drainage**  **fluid** | **SSI** |  | | **Drainage**  **fluid** | | **SSI** |  |  |
| Amikacin | S | S |  |  | | | S | S |  | S | S |  | * | * |  | S | S |  | | | S | S |  | |
| Ampicilin | * | * |  |  | | | R | R |  | * | * |  | S | S |  | R | R |  | | | * | * |  | |
| Aztreonam | **S** | **I** |  |  | | | S | S |  | **S** | **R** |  | * | * |  | S | S |  | | | S | S |  | |
| Cefaclor | * | * |  |  | | | R | R |  | * | * |  | * | * |  | R | R |  | | | * | * |  | |
| Cefazolin | * | * |  |  | | | R | R |  | * | * |  | * | * |  | R | R |  | | | * | * |  | |
| Cefcapene pivoxil | * | * |  |  | | | **R** | **S** |  | * | * |  | * | * |  | I | I |  | | | * | * |  | |
| Cefepime | **S** | **I** |  |  | | | S | S |  | S | S |  | * | * |  | S | S |  | | | S | S |  | |
| Cefmetazole | * | * |  |  | | | R | R |  | * | * |  | * | * |  | R | R |  | | | * | * |  | |
| Cefotaxime | S | R |  |  | | | S | S |  | S | S |  | * | * |  | S | S |  | | | * | * |  | |
| Cefotiam | * | * |  |  | | | **R** | **I** |  | * | * |  | * | * |  | R | R |  | | | * | * |  | |
| Ceftazidime | S | R |  |  | | | S | S |  | * | * |  | * | * |  | S | S |  | | | S | S |  | |
| Ceftriaxone | * | * |  |  | | | S | S |  | * | * |  | * | * |  | S | S |  | | | * | * |  | |
| Ciprofloxacin | S | S |  |  | | | S | S |  | S | S |  | * | * |  | S | S |  | | | S | S |  | |
| Doripenem | S | S |  |  | | | * | * |  | S | S |  | * | * |  | * | * |  | | | S | S |  | |
| Erythromycin | * | * |  |  | | | * | * |  | * | * |  | R | R |  | * | * |  | | | * | * |  | |
| Flomoxef | * | * |  |  | | | I | I |  | * | * |  | * | * |  | R | R |  | | | * | * |  | |
| Fosfomycin | R | I |  |  | | | S | S |  | R | R |  | * | * |  | I | I |  | | | R | R |  | |
| Gentamicin | S | S |  |  | | | S | S |  | S | S |  | * | * |  | S | S |  | | | S | S |  | |
| Imipenem | S | S |  |  | | | **S** | **I** |  | S | S |  | S | S |  | S | S |  | | | S | S |  | |
| Levofloxacin | S | S |  |  | | | S | S |  | S | S |  | S | S |  | S | S |  | | | S | S |  | |
| Linezolid | * | * |  |  | | | * | * |  | * | * |  | S | S |  | * | * |  | | | * | * |  | |
| Meropenem | S | S |  |  | | | S | S |  | S | S |  | * | * |  | S | S |  | | | S | S |  | |
| Minocycline | * | * |  |  | | | S | S |  | * | * |  | I | I |  | S | S |  | | | * | * |  | |
| Piperacilin | **S** | **R** |  |  | | | S | S |  | **S** | **I** |  | * | * |  | S | S |  | | | S | S |  | |
| Rifampicin | * | * |  |  | | | * | * |  | * | * |  | S | S |  | * | * |  | | | * | * |  | |
| Sulbactam/Ampicilin | * | * |  |  | | | S | S |  | * | * |  | * | * |  | R | R |  | | | * | * |  | |
| Sulbactam/Cefoperazone | **S** | **R** |  |  | | | S | S |  | S | S |  | * | * |  | S | S |  | | | S | S |  | |
| Sulfamethoxazole/Trimethoprim | * | * |  |  | | | S | S |  | * | * |  | * | * |  | S | S |  | | | * | * |  | |
| Tazobactam/Piperacillin | **S** | **R** |  |  | | | S | S |  | S | S |  | * | * |  | S | S |  | | | S | S |  | |
| Teicoplanin | * | * |  |  | | | * | * |  | * | * |  | S | S |  | * | * |  | | | * | * |  | |
| Tobramycin | S | S |  |  | | | * | * |  | S | S |  | * | * |  | * | * |  | | | S | S |  | |
| Vancomycin | * | * |  |  | | | * | * |  | * | * |  | S | S |  | * | * |  | | | * | * |  | |

I, in-between; S, sensitive; R, resistant; *, not examined
